# Supplementary material for: Vat-Mediated Mucus Penetration Enables Genotoxic Activity of pks+ Escherichia coli
Source: Int J Mol Sci. 2025 Jun 3;26(11):5353. doi: 10.3390/ijms26115353 (PMC12155319; doi:10.3390/ijms26115353)
Supplement: Supplementary file 1 [file ijms-26-05353-s001.zip › ijms-3638582-supplementary.pdf]

Supplemental data

Table S1. Clinical strains used in this study

Strains	Description	Strain reference
POP198	Clinical strain isolated from patient harbouring *pks* island (colibactin production) and *vat* (Vat production).	*pks+vat+*
POP198Δ*vat*	POP198 strain deleted for *vat* gene	*pks+vat-*
POP198Δ*vat* + pBAD*vat*	POP198 strain deleted for *vat* gene and trans-complemented with pBAD33.1 with *vat* gene.	*pks+vat-* *+pBAD-vat*
11G5	Clinical strain isolated from patient harbouring *pks* island (colibactin production) and *vat* (Vat production).	*pks+vat+*
11G5Δ*vat*	11G5 strain deleted for *vat* gene.	*pks+vat-*
11G5Δ*vat* + pBAD*vat*	11G5 strain deleted for *vat* gene and trans-complemented with pBAD33.1 with *vat* gene.	*pks+vat-* *+pBAD-vat*

Table S2. Primers, plasmids and probes used in this study

Primer	Sequence (5'→ 3')	Product size	Use	Reference
Vat-F	GGGGCGCCGGATGAGGCATTA	204 bp	Detection of *vat*	This study
Vat-R	CCTCTTTTGGAGAGGCCGGCGACAGT			
Hbp/tsh-F	ACTATTCTCTGCAGGAAGTC	824 bp	Detection of *hbp/tsh*	[36]
Hbp/tsh-R	CTTCCGATGTTCTGAACGT			
Pic-F	ACTGGATCTTAAGGCTCAGG	411 bp	Detection of *pic*	[36]
Pic-R	TGGAATATCAGGGTGCCACT			
ClbQ-F	GACGGCATCCACCATCGTAA	230 bp	Detection of *clbQ*	[30]
ClbQ-R	TAAATGGCGTAGCGTGCTGT			
Sp_cat_F	TACCATATTGTGGATCGTAATGAACACA GTTCATCTGATCTCCACACACCCACTGGC ATTTAATAACGCGTC	1 100 pb	Spectinomycin resistance cassette amplification	This study
Sp_cat_R	AGTTGCGCCATAATCGGCGGCATTCAGC TTATGAAATGTCAGGTCATTCCTTAATCA CTTTACTTTTATC			
VATForXba	GCTCTAGAATGAATAAAATATACGCTCT	4,149 bp	*vat* cloning in pBAD33.1	This study
VATRevHind	GCAAGCTTTCAGTGGTGATGGTGATGAT GGAATGAATAACGAATATTAG			
Pbad33.1_CmF_F	GGGTACACTTACCTGTGATCGGCACGTA AGAGGTTCC	5.434 bp	pBAD33.1 amplification	This study
Pbad33.1_CmF_R	ATTTTCCACCCGGGTTTACGCCCCGCCCT GCCACTCATCG		pBAD33.1 amplification	

Plasmid	Description	Use	Reference
pBAD33.1	pBAD cloning vector with an arabinose inducible promotor; chloramphenicol resistant.	Clonage of *vat* gene.	[35]
pKOBEG	pBAD cloning vector harboring a λ phage redγβα operon; chloramphenicol resistant.	Deletion of *vat* gene.	[35]

Probes	Sequence (5'→ 3')	Use	Reference
Cy3-*E. coli*	GCAAAGGTATTAACTTTACTCCCTTCCTCC	Localisation of *E. coli* bacteria.	[38]

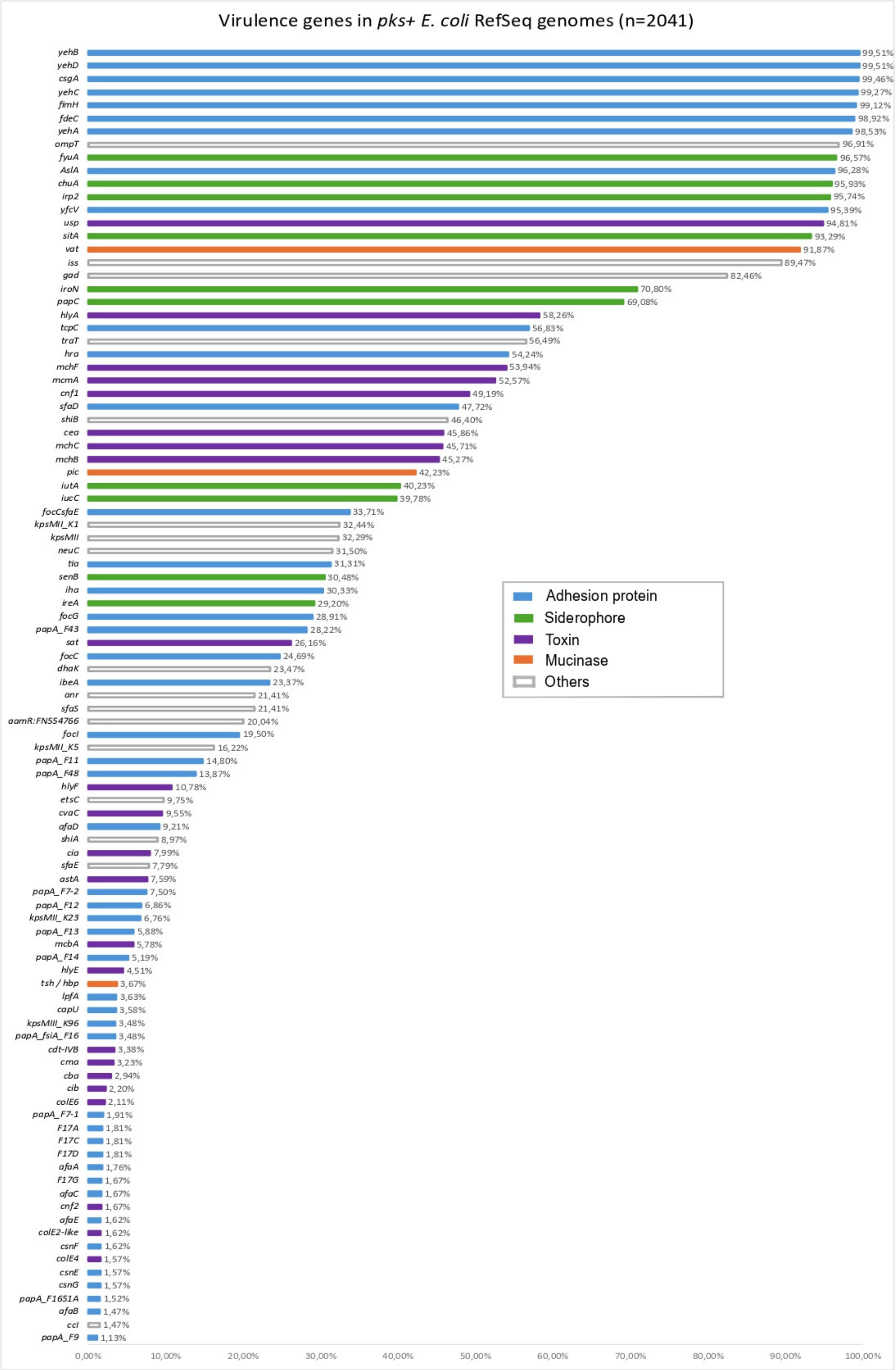
Virulence genes in pks+ E. coli RefSeq genomes (n=2041)
yehB 99,51%
yehD 99,51%
csgA 99,46%
yehC 99,27%
fimH 99,12%
fdeC 98,92%
yehA 98,53%
ompT 96,91%
fyuA 96,57%
AslA 96,28%
chuA 95,93%
irp2 95,74%
yfcV 95,39%
usp 94,81%
sitA 93,29%
vat 91,87%
iss 89,47%
gad 82,46%
iroN 70,80%
papC 69,08%
hlyA 58,26%
tcpC 56,83%
traT 56,49%
hra 54,24%
mchF 53,94%
mcmA 52,57%
cnf1 49,19%
sfaD 47,72%
shiB 46,40%
cea 45,86%
mchC 45,71%
mchB 45,27%
pic 42,23%
iutA 40,23%
iucC 39,78%
focCsfaE 33,71%
kpsMII_K1 32,44%
kpsMII 32,29%
neuC 31,50%
tia 31,31%
senB 30,48%
iha 30,33%
ireA 29,20%
focG 28,91%
papA_F43 28,22%
sat 26,16%
focC 24,69%
dhaK 23,47%
ibeA 23,37%
anr 21,41%
sfaS 21,41%
aamR:FN554766 20,04%
focI 19,50%
kpsMII_K5 16,22%
papA_F11 14,80%
papA_F48 13,87%
hlyF 10,78%
etsC 9,75%
cvaC 9,55%
afaD 9,21%
shiA 8,97%
cia 7,99%
sfaE 7,79%
astA 7,59%
papA_F7-2 7,50%
papA_F12 6,86%
kpsMII_K23 6,76%
papA_F13 5,88%
mcbA 5,78%
papA_F14 5,19%
hlyE 4,51%
tsh / hbp 3,67%
lpfA 3,63%
capU 3,58%
kpsMIII_K96 3,48%
papA_fsiA_F16 3,48%
cdt-IVB 3,38%
cma 3,23%
cba 2,94%
cib 2,20%
colE6 2,11%
papA_F7-1 1,91%
F17A 1,81%
F17C 1,81%
F17D 1,81%
afaA 1,76%
F17G 1,67%
afaC 1,67%
cnf2 1,67%
afaE 1,62%
colE2-like 1,62%
csnF 1,62%
colE4 1,57%
csnE 1,57%
csnG 1,57%
papA_F1651A 1,52%
afaB 1,47%
ccl 1,47%
papA_F9 1,13%
0,00%
10,00%
20,00%
30,00%
40,00%
50,00%
60,00%
70,00%
80,00%
90,00%
100,00%
Adhesion protein
Siderophore
Toxin
Mucinase
Others

Figure S1: Virulence genes found in *pks*+ *E. coli* genome.

Using RefSeq database (v1.214 databases), we determined the number of *pks*+ *E. coli*. For that, we blasted *E. coli* genomes against *clbQ* gene which is always present inside the *pks* island (*clbQ* is required for the production of colibactin). Among the 2041 *pks*+ *E. coli* genome-selected, virulence genes were identified using the VirulenceFinder 2.0 tool. The figure shows the virulence genes most frequently found (>1%) in *pks*+ *E. coli*. Virulence genes were classified in five proteins families in link with their biological functions.

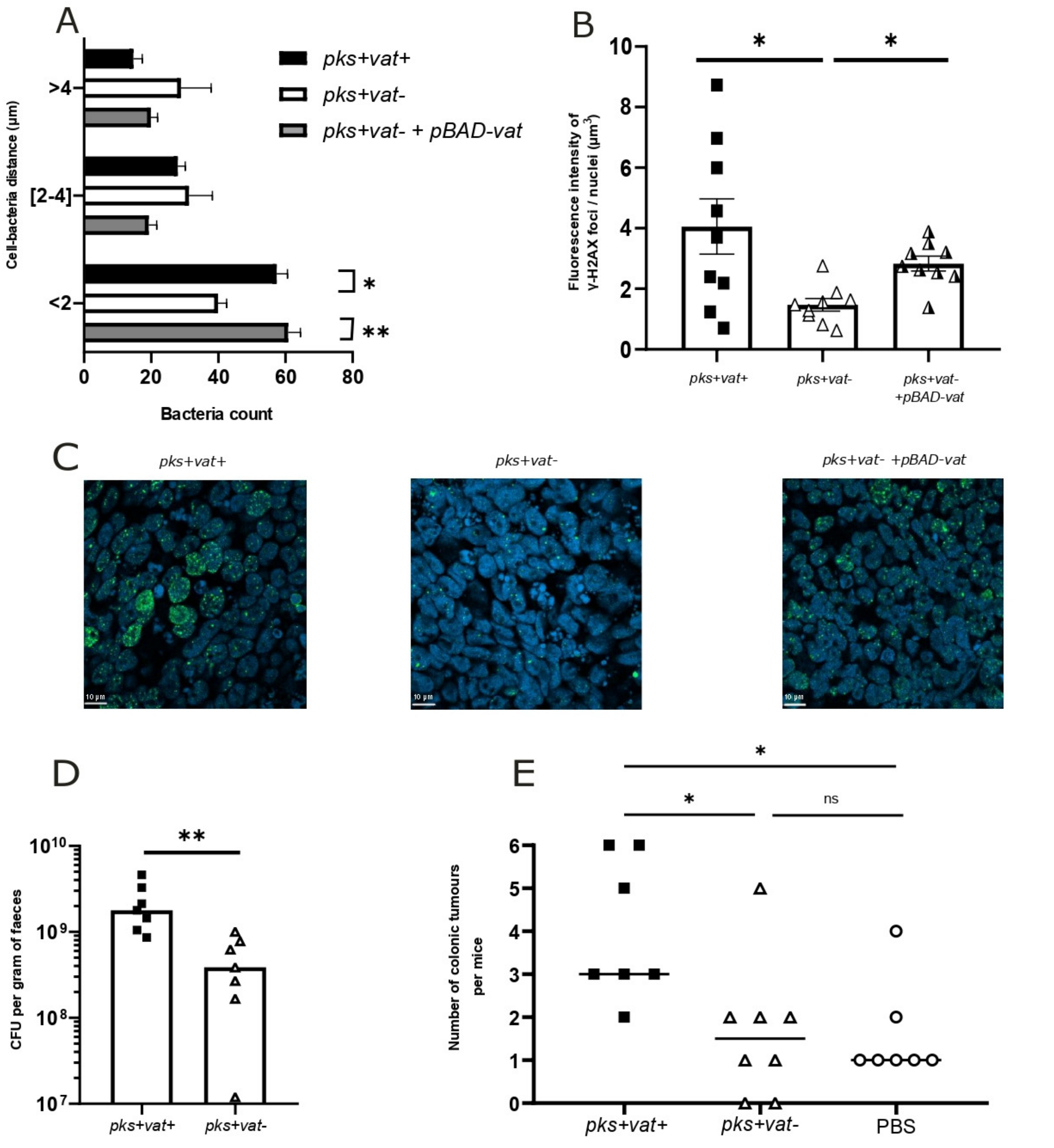

Figure S2: Vat favours mucus-crossing, gut colonization, genotoxicity of *pks+ E. coli* 11G5 and increases colonic tumours numbers in *Apc$^{Min/+}$* mice. (**A**) Mucus-producing HT29-16E cells were infected for 45 minutes with a Multiplicity of Infection of 100. Cells were stained by Hoechst (blue), mucus by WGA-leptin (red) and *pks+ E. coli* by fluorescence *in situ* hybridization using Cy3-*pks+ E. coli* probe. For each replicated, three representative fields were analysed, and experiments were performed 3 times. Using Imaris software, the bacteria to cell distance was determined for each field and bacteria was counted in each 2 µm layer. Data are presented as means ± SEMs. (**B-C**) Mucus-producing HT29-16E cells were infected for 90

minutes with a Multiplicity of Infection of 100. For each replicated, three representative fields were analysed, and experiments were performed 3 times. Phosphorylated γ-H2AX foci fluorescence intensity was determined by immunofluorescence 24 hours post-infection and expressed on the nuclei volume (Hoechst staining). Data are presented as means ± SEMs. Statistical comparisons were carried out by one-way Kruskal–Wallis's test nonparametric for followed by the Dunn post's tests after normality testing (*$P < 0.05$, **$P < 0.01$). (**D-E**) *Apc*$^{Min/+}$ mice were orally administered 10^9 colony-forming units *E. coli pks+vat+, E. coli pks+vat-* mutant, or with PBS. (**D**) Bacterial colonization in the stools of mice at the final point of experiment on day-56 post-infection. The data points represent actual values for each individual mouse, and the bars indicate medians. (**E**) The number of colorectal tumours by mouse was determined using a dissecting microscope. Data are presented as medians. Statistical comparisons were carried out by one-way Kruskal–Wallis's test nonparametric for three groups mice or Mann-Whitney test analysis for two groups mice followed by the Dunn post's tests after normality testing (*$P < 0.05$, **$P < 0.01$, ns = not significant).

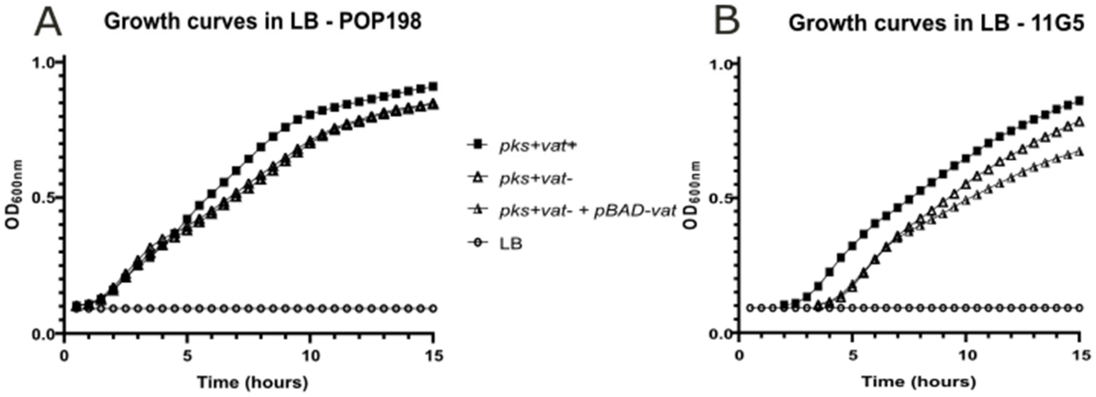

Figure S3: Growth curves of POP198 (A) and 11G5(B) strains, isogenic mutants and trans-complemented strains in Luria-Bertani broth (LB) media. Each strain was cultured overnight in LB before dilution to OD_{600}=0.1. Optic Density was measured at 600 nm by microplates multimodes SPARK® from 15 hours. Experiments were repeated three time with three replicates for each.
